# Premorbid Alterations of Spontaneous Brain Activity in Elderly Patients With Early Post-operative Cognitive Dysfunction: A Pilot Resting-State Functional MRI Study

**DOI:** 10.3389/fneur.2019.01062

**Published:** 2019-10-09

**Authors:** Xixue Zhang, Hui Li, Yating Lv, Zhenghong Zhu, Xiaoyong Shen, Qi Lu, Wei Wang, Zhaoxin Wang, Zhaoshun Jiang, Lvjun Yang, Guangwu Lin, Weidong Gu

**Affiliations:** ^1^Department of Anesthesiology, Huadong Hospital, Fudan University, Shanghai, China; ^2^Shanghai Key Laboratory of Clinical Geriatric Medicine, Shanghai, China; ^3^Institutes of Psychological Sciences, Hangzhou Normal University, Hangzhou, China; ^4^Zhejiang Key Laboratory for Research in Assessment of Cognitive Impairments, Hangzhou, China; ^5^Department of Thoracic Surgery, Huadong Hospital, Fudan University, Shanghai, China; ^6^Department of General Surgery, Huadong Hospital, Fudan University, Shanghai, China; ^7^Shanghai Key Laboratory of Brain Functional Genomics, Key Laboratory of Brain Functional Genomics, Ministry of Education, Shanghai, China; ^8^Institute of Cognitive Neuroscience, East China Normal University, Shanghai, China; ^9^Department of Radiology, Huadong Hospital, Fudan University, Shanghai, China

**Keywords:** resting-state fMRI, pre-operative changes in brain, cognitive decline, early post-operative period, functional connectivity, regional homogeneity

## Abstract

**Background:** Elderly patients with pre-existing cognitive impairment are susceptible to post-operative cognitive dysfunction (POCD). In this study, we investigated whether there is pre-existing local homogeneity and functional connectivity alteration in the brain before surgery for POCD patients as compared to that in non-POCD patients.

**Methods:** Eighty elderly patients undergoing major thoracic or abdominal surgeries were recruited. Resting-state functional MRI was scanned at least 1 day before surgery. Neuropsychological tests (NPTs) were performed before surgery and at discharge, respectively. Pre-operative regional homogeneity (ReHo) and resting-state functional connectivity (RSFC) were compared between POCD patients and non-POCD patients, respectively. Partial correlation between NPTs and ReHo or RSFC was analyzed by adjusting for confounding factors.

**Results:** Significant difference (*P* < 0.001, Gaussian Random Field (GRF) correction which is a multiple comparisons correction method at cluster level, cluster size > 49) in ReHo between POCD patients and non-POCD patients was detected in right hippocampus/parahippocampus. Pre-operative RSFC between right hippocampus/parahippocampus and right middle/inferior temporal gyrus increased in POCD patients (*P* < 0.001, GRF correction for multiple comparisons) when compared with that in non-POCD patients.RSFC significantly correlated with composite *Z*-score (*r* = 0.46, 95% CI [0.234, 0.767], *P* = 0.002) or Digit Symbol Substitution Test *Z*-scores (*r* = 0.31, 95% CI [0.068, 0.643], *P* = 0.046) after adjusting for confounding factors.

**Conclusions:** The results suggest that premorbid alterations of spontaneous brain activity might exist in elderly patients who develop early POCD. The neural mechanism by which patients with pre-operative abnormal spontaneous activity are susceptible to POCD requires further study.

## Introduction

The incidence of early post-operative cognitive dysfunction (POCD) is 25.8% in those who underwent major non-cardiac surgical procedures (1). There is accumulating evidence suggesting that pre-existing cognitive impairment (PreCI) is prevalent in geriatric elective surgical patients (2), and PreCI is likely a good predictor of cognitive dysfunction after surgery (3). Also, patients with Alzheimer's disease neuropathology (lower pre-operative cerebrospinal fluid Aβ1–42) even in the absence of clinically detectable symptoms may be susceptible to POCD (4). These findings indicate that pre-operative asymptomatic cognitive decline or neuropathology exists in patients who develop POCD. Numerous studies support the assertion that cognitive function is closely related to individual spontaneous brain activity patterns detected by functional magnetic resonance imaging (fMRI) (5, 6). Therefore, whether POCD patients also have different pre-operative spontaneous brain activity is an interesting question.

In recent, it has been demonstrated that pre-operative neuroanatomical changes, including reduced gray matter of bilateral medial temporal lobe (MTL), white matter lesions, and greater pre-operative volumes of leukoaraiosis/lacunae and cerebrovascular damage are associated with POCD (7). However, the study evaluating the predictive value of pre-operative brain functional alterations is limited. To the best of our knowledge, there is only one study that examined relationships between post-operative changes in resting-state functional connectivity (RSFC) in default mode network (DMN) regions and POCD after cardiac surgery (8). The abnormalities in pre-operative brain function and their relationship with POCD are still unclear.

Resting-state functional magnetic resonance imaging (RsfMRI) is a promising tool to investigate functional alterations of the human brain *in vivo* (9). Regional homogeneity or functional connectivity are usually aberrant in patients with cognitive impairment. Regional homogeneity (ReHo) and RSFC are two frequently-used methods to characterize local and global blood oxygenation level-dependent (BOLD) signal synchronization. ReHo is proposed as a voxel-wise measure of the synchronization of the time series of neighboring voxels (10), which has been employed to investigate the disorders with cognitive alterations, such as psychiatric diseases (schizophrenia, attention deficit hyperactivity disorder) (11, 12), neurological disorders (Alzheimer's disease and Parkinson's disease) and healthy aging (13–15). RSFC is defined as the temporal correlation of BOLD signal among spatially remote brain areas (16). Previous studies have demonstrated the correlation between RSFC and cognitive performance. Therefore, RSFC alterations might serve as indicators for the gradual cognitive dysfunction in the early stage of Alzheimer's disease (17) and for the reorganization of the resting-state network in healthy aging (18).

In this study, we investigated whether there is pre-existing local homogeneity and functional connectivity alteration in the brain before surgery for POCD patients as compared to that in non-POCD patients. We aimed to explore the altered brain regions by using ReHo analysis, and then these brain regions were selected as regions of interest (ROI) for RSFC analysis. Our first goal was to investigate the differences in pre-operative RsfMRI manifestations between POCD patients and non-POCD patients. The second goal was to explore the correlation between pre-operative spontaneous brain activity and NPTs in all patients (both POCD and non-POCD patients).

## Materials and Methods

### Protocol Approvals, Registrations, and Patient Consents

**Figure 1:**
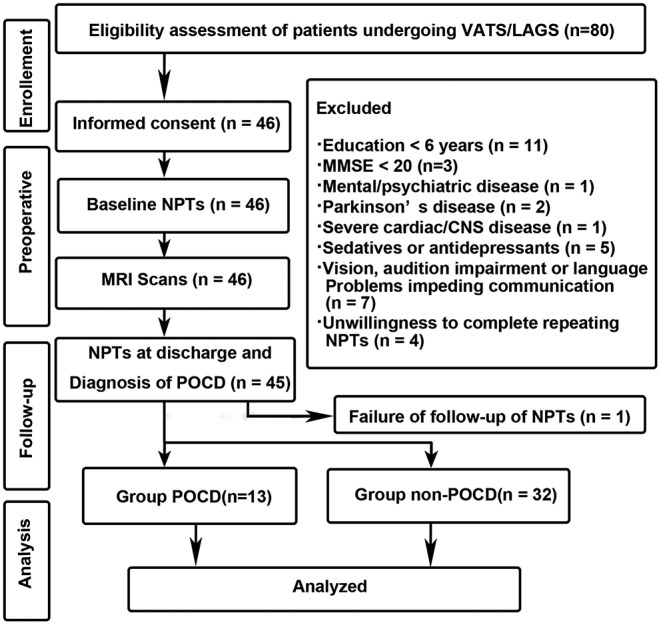
Study design and flow-chart. VARS, Video-assisted Thoracoscopic Surgery; LAAS, Laparoscopy-assisted Abdominal Surgery; NPTs, neuropsychological tests.

This study has been approved by the Ethics Committee of Huadong Hospital affiliated with Fudan University with the Approved Number of 20150056. This study was registered before patient enrollment at http://www.chictr.org.cn with the identifier of ChiCTR-DCD-15006096 on 16th March 2015. Written informed consent was obtained from every participant after full explanation of the protocol. Authors followed the Declaration of Helsinki principles. The flow-chart of the study design is listed as follows (Figure 1).

### Participants

Patients were recruited from 30th March 2015 to 31st May 2016. In order to correct for practice effect, 20 community individuals, with age and education-matched to the patient group, were selected from the spouse of surgical inpatients as volunteers who did NOT participate in this study. All these volunteers were given the Mini-Mental State Examination and received a score of 20 or higher. All of the volunteers completed the same neuropsychological tests as those performed by the patients.

The inclusion criteria for the patient group were as follows: (1) patients prepared to undergo major thoracic or abdominal surgeries; (2) age > = 60 years; (3) American Society of Anesthesiologists (ASA) Physical Status Classification wasI-II; (4) Right handedness.

The exclusion criteria were: (1) education < 6 years; (2) pre-operative Mini-Mental State Examination < 20; (3) pre-existing mental and/or psychiatric disease; (4) Parkinson's disease; (5) history of cardiac and/or central nervous system vascular disease; (6) history of cardiac and cranial surgeries; (7) taking sedatives or antidepressants in the last year; (8) alcohol or drugs abuse; (9) severe hepatic or renal dysfunction; (10) vision, audition impairment or language troubles impeding communication; (11) situations unsuitable for an MRI scan (claustrophobia); (12) unwillingness to complete repeat NPTs.

### Study Design

Patients were recruited 1–3 days before surgery. The RsfMRI scan and the baseline NPTs were performed at least 1 day before surgery. Post-operative NPTs were performed at discharge (19, 20) and 3-months after surgery. We gave calls to all patients and invited them to revisit to our hospital 3-months after surgery. All patients were encouraged to participate in this study by giving some little gifts when the follow-ups were completed. The healthy volunteers completed NPTs at an equivalent interval. The performance of NPTs from healthy volunteers was used to construct a *Z*-scores (21). After calculating the *Z*-scores and composite Z-score, POCD was diagnosed according to the method adopted by Moller et al. (1). Subsequently, patients were divided into POCD group and non-POCD group. The demographics, RsfMRI data, NPTs results, perioperative data were collected and analyzed.

### Anesthesia Protocols

General anesthesia induction consisted of propofol, rocuronium, sufentanil through a central intravenous catheter. Benzodiazepines were avoided because of their potential effects on cognitive ability. Parameters of mechanical ventilation were adjusted to maintain the end-tidal carbon dioxide of 35 ± 5 mmHg. Anesthesia maintenance was comprised of inhaled sevoflurane or continuous infusion of propofol and remifentanil. Intravenous rocuronium and sufentanil were administered intermittently. Bispectral index (BIS) was kept between 60 and 40 to ensure appropriate anesthesia depth. Intravenous patient-controlled analgesia (PCA) was used to keep the visual analog scale (VAS) score < 3 after surgery, and the PCA formula contained sufentanil and ketorolac tromethamine.

### Neuropsychological Tests and Definition of POCD

NPTs were performed at two time points for all patients and healthy volunteers: (1) Baseline measurement: at least 1 day before surgery; (2) At discharge.

The NPTs battery consisted of six tests: Mini-Mental State Examination, Verbal Fluency Test, Digit Span (Forward and Backward), Digit Symbol Substitution Test, and Trail Making Test part A. Mini-Mental State Examination is an appropriate tool for dementia screening, which can reach good diagnostic accuracy with 0.81 sensitivity (95% CI, 0.78–0.84) and 0.89 specificity (95% CI, 0.87–0.91) (22). Verbal Fluency Test is a semantic memory test, in which participants were asked to speak out “vegetable” as many as possible in 60 s, and the correct score was documented by voice recording to avoid incorrect records. The Digit Span tests were used to measure working memory and attention. During Digit Span tests, patients were required to repeat a series of numbers (forward or backward) in a randomized sequence. The Digit Symbol Substitution Test is a paper-pencil test which examines attention, executive function as well as working memory. During the test, patients were given randomly ordered numbers paired with empty boxes which were required to fill as many as possible in 90 s. The Trail Making Test part A is designed to detect the ability of visual search speed as well as visual-motor skills, in which the patients connected 25 numbers from 1 to 25. The amount of time consumed (seconds) was recorded as a measure of performance. All of the NPTs were administered to all patients and volunteers by a skilled researcher (H. Li) in a quiet room.

A *Z*-scores was calculated for every single test according to the method recommended by the International Study of Post-operative Cognitive Dysfunction (ISPOCD1) to delineate patients' post-operative cognitive alteration (1). Considering the practice effects of repeated tests, we enrolled 20 healthy volunteers who were age- and education-matched with the patients. Healthy volunteers completed the NPTs mentioned above at the same interval. The difference of patients' pre-operative- and post-operative-score was named Δx, and the counterpart of the volunteer group was calculated to be Δxc (mean value for difference in the volunteer group), the standard deviation (SD) of Δxc was computed to be SD(Δxc), so the *Z*-score could be built as follow:

$$\text{Z= }\frac{\left(\Delta \text{x}-\Delta \text{xc}\right)}{\text{SD}\left(\Delta \text{xc}\right)}$$

A positive sign *Z*-score represented deterioration in the corresponding test; on the contrary, a negative *Z*-score meant the improvement of cognition. A sum of a subject's all six *Z*-scores was then divided by the standard deviation for the changes of test results in the volunteer group, SD(ΔXc), in this way, a composite *Z*-score can be constructed. POCD was defined as at least two of the NPTs *Z*-scores were > 1.96 or the composite *Z*-score was > 1.96.

### MRI Scans

MRI data were acquired using a 3 Tesla MRI scanner (SIEMENS Skyra) at Huadong Hospital affiliated to Fudan University, Shanghai, China. During the MRI data acquisition, participants were instructed to keep awake, relax with their eyes closed and remain motionless as much as possible. For each patient, the MR scanning protocol included the following sessions: (1) RsfMRI data was acquired using an echo-planar imaging sequence: 33 axial slices, slices of thickness = 4 mm with 0 mm gap, TR = 3,000 ms, TE = 30 ms, voxel size = 3.4 × 3.4 mm × 4.0 mm, flip angle = 90°. In this scan, 120 volumes were obtained; (2) 3D high resolution T1-weighted anatomical images were acquired using a 3D-MPRAGE sequence: 176 sagittal slice, TR = 1,900 ms, TE = 3.57 ms, voxel size = 1.0 × 1.0 × 1.0 mm, flip angle = 9°.

### Data Pre-processing

RsfMRI data was processed using DPABI (23), including (1) discarding the first five volumes of functional images to make the longitudinal magnetization reach steady state and to let the participant get used to the scanning noise; (2) head motion correction; (3) spatial normalization: 3D T1 images were aligned to individual averaged functional image and subsequently spatially normalized to the MNI template by using the deformation field from segmentation analysis; (4) removing the linear trend of the time course; (5) regressing out the head motion effect [using Friston 24 parameter (24)] from the fMRI data; and (6) band-pass (0.01– 0.08 Hz) filtering. No participant's head motion exceeded 3.0 mm of maximal translation (in any direction of x, y, or z) or 3.0° of maximal rotation throughout scanning.

### Regional Homogeneity (ReHo)

ReHo method proposed by Zang et al. was used to analyze fMRI data (13), in which Kendall's coefficient of concordance (KCC) was applied to quantify the functional synchronization of the time courses of neighboring voxels as follows:

$$W=\frac{\sum {\left(R_{i}\right)}^{2}-n{\left(R\right)}^{2}}{\frac{1}{12}K^{2}\left(n^{3}-n\right)}$$

where *W* is the KCC calculated from given voxels, ranging from 0 to 1; Ri is referred to the sum rank of the *i*th time point; $R=\frac{\left(n+1\right)K}{2}$ is the R*i*'s mean value; *K* is the number of time series within a measured cluster (*K* = 7, 19, and 27, respectively, 27 in the current study); and *n* is the number of ranks. All the ReHo maps' results were smoothed by an isotropic Gaussian Kernel with 6 mm full width at half-maximum (FWHM).

### Functional Connectivity

The clusters which obtained from group difference of ReHo analysis were picked as the ROIs for functional connectivity (FC) analysis. For each ROI, the Pearson correlation analysis was calculated between the averaged time courses of the ROI and the time course of all other voxels in the brain. The resultant correlation coefficients were transformed to z-value using Fisher's transformation.

### Statistical Analysis

Standard Chi-square statistics or Fisher's exact test was used to analyze categorical variables. Continuous-valued data were analyzed either using two-sample *t*-test and were presented as the mean and standard deviation (SD) or Mann–Whitney *U*-test when a data distribution was assumed to be abnormal. Discrete data were analyzed with the Mann-Whitney *U*-test and presented as the median and interquartile range (IQR). A two-sided *P* < 0.05 was considered significant. All analyses were performed using SPSS 18.0 (IBM, Armonk, NY, USA), Matlab 2014a (Mathworks, Massachusetts, USA) or Statistical Parametric Mapping (SPM12).

Two-sample *t*-test was performed to detect the differences of ReHo or ReHo-seeded FC between the POCD group and the non-POCD group in SPM12. In order to correct the possible confounders including education, sex, and smoking, we set these variables as covariates when two-sample *t*-test was performed in SPM 12. The resultant T-map was threshold with P <0.001, corrected for multiple comparisons using the Gaussian random field (GRF) method to minimize type I error (25).

Partial correlation analysis was used to assess the correlations between brain functional indices (ReHo or ReHo-seeded FC) neuropsychological scale (Mini-Mental State Examination, Verbal Fluency Test, Digit Span Forward, Digit Span Backward, Digit Symbol Substitution Test, and Trail Making Test part A) scores after adjusting for potential confounding factors. The correlations were considered significant at a threshold of *P* < 0.05.

The initial sample size of our study was estimated based on the available studies and the pragmatics of recruitment and the necessities for examining feasibility (26). A power analysis was performed at the end of the study and determined a statistical power of 0.99 for the primary outcome (RSFC).

## Results

### Demographics, Clinical Characteristics

One patient was excluded from the final analysis because of worsening vision during the post-operative period and subsequent failure of finishing several items of the NPTs. Forty-five patients completed both pre-operative MRI scan and NPTs follow-up. The demographics of the 45 patients are shown in the Table 1. Thirteen patients were diagnosed with POCD, and the incidence of POCD was 26.7% at discharge. The average age of the patients was 65 ± 5 years in both the POCD and non-POCD groups. The education duration in POCD patients (6.0, IQR: 6.0–10.5) was significantly lower than that in non-POCD patients (9.0, IQR: 9.0–12.0, *P* = 0.006). The proportion of male patients in the POCD group (12/1) was significantly greater than that in the non-POCD group (14/18, *P* = 0.003). There were no differences in hospital stay and intraoperative data between the POCD group and the non-POCD group (Table 1). Sixteen patients (34.8%) did not revisit our hospital at 3 month after surgery.

**Table 1:** Demographics and clinical characteristics.

	**POCD (*n* = 13)**	**Non-POCD (*n* = 32)**	***P-*value**
**Demographics**			
Age (years), mean (SD)	65 (5)	65 (5)	0.69
Education, median (IQR)	6.0 (6.0–10.5)	9.0 (9.0–12.0)	0.006
BMI, median (IQR)	20.8 (18.3–26.1)	21.7 (20.5–24.4)	0.32
**Gender**			
Female/Male	1/12	18/14	0.003
Smoking (Yes/No)	7/6	6/26	0.030
**Comorbidities (Yes/No)**			
Cardiovascular	5/8	11/21	1.00
Anemia	4/8	8/24	0.71
Hepatorenal dysfunction	4/9	7/25	0.70
Surgical history (Yes/No)	6/7	14/17	0.95
Surgery type (Thoracic/Abdominal)	10/3	25/7	1.00
**Intraoperative conditions**			
Surgical duration (min), median (IQR)	125.0 (109.5–175.0)	95.5 (66.0–143.5)	0.08
Anesthesia duration (min), median (IQR)	161.0 (137.0–220.0)	137.5 (105.8–218.3)	0.18
Propofol (mg), median (IQR)	200.0 (65.0–553.5)	233.5 (50.0–462.3)	0.72
Sevoflurane(MAC-hours), median (IQR)	1.90 (0.6–2.8) (0.0–41.2)	1.01 (0.0–2.7) (0.0–22.7)	0.16
Sufentanil (μg), mean (SD)	30 (14)	27 (9)	0.36
Remifentanil (μg), median (IQR)	1,342.0 (1,183.0–2,000.0)	1,191.5 (725.0–2,000.0)	0.40
Ringers (mL), mean (SD)	1,200 (488)	1,063 (419)	0.43
Colloid (mL), median (IQR)	500.0 (500.0–1,000.5)	100.0 (0.0–500.0)	0.30
Urine (mL), median (IQR)	100.0 (0.0–575.0)	100.0 (100.0–300.0)	0.17
**Hospital stay (days)**			
median (IQR)	14.0 (10.5–16.5)	12.0 (9.0–13.0)	0.21

*BMI, body mass index; SD, standard deviation; IQR, interquartile range*.

### Alterations of NPTs Performance (*Z*-Scores)

There were no significant differences in the baseline NPTs performance between the POCD group and the non-POCD group. However, Verbal Fluency Test, Digit Span Forward, Digit Span Backward, Digit Symbol Substitution Test, and composite *Z*-scores in the post-operative period were significantly different between POCD patients and non-POCD patients (Supplemental Table 1).

### ReHo Differences at the Group Level

**Figure 2:**
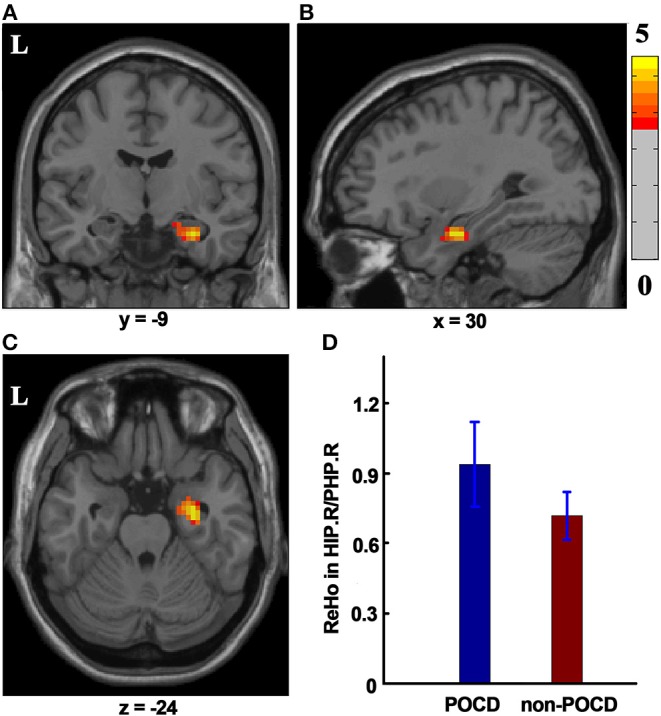
The ReHo difference between the POCD group and the non-POCD group. Warm colors in **(A–C)** represent coronal, sagittal, and axial view of significantly increased ReHo value in the HIP.R/PHP.R in the POCD group (*P* < 0.001, GRF corrected, cluster size >49). Peak MNI coordinate: *x* = 30, *y* = −9, *z* = −24, 61 voxels in total. **(D)** ReHo value in the POCD group significantly increased as compared to that in the non-POCD group. ReHo, regional homogeneity. L, left; HIP.R/PHP.R, right hippocampus and right parahippocampus; POCD, post-operative cognitive dysfunction; GRF, Gaussian Random Field; MNI, Montreal Neurological Institute.

The significant difference (*P* < 0.001, GRF Correction, cluster size > 49) in ReHo was detected between the POCD group and the non-POCD group. The ReHo value displayed in the right hippocampus/parahippocampus in the POCD group is higher than that in the non-POCD group (Figure 2).

### RSFC Differences at a Group Level

**Figure 3:**
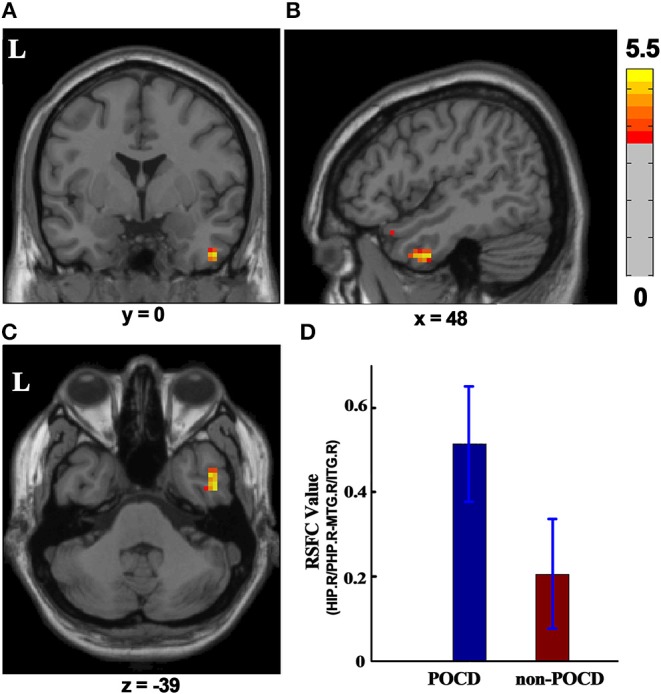
RSFC difference between the POCD group and the non-POCD group (*P* < 0.001, GRF corrected, cluster size > 26). Warm colors in **(A–C)** represent coronal, sagittal, and axial view of significantly increased HIP.R/PHP.R-seeded RSFC in MTG.R/ITG.R in the POCD group, respectively. Peak MNI coordinate: *x* = 48, *y* = 0, *z* = −39, 41 voxels in total. **(D)** HIP.R/PHP.R-seed RSFC in MTG.R/ITG.R in the POCD group and in the non-POCD group. RSFC, resting-state functional connectivity; MNI, Montreal Neurological Institute; L, left; HIP.R/PHP.R, right hippocampus and right parahippocampus; MTG.R/ITG.R, right middle temporal gyrus and right inferior temporal gyrus; POCD, post-operative cognitive dysfunction.

Cluster in the right hippocampus/parahippocampus with increased ReHo value was selected as ROI, which was used to compute functional connectivity by comparing the seed time series with every other voxel of the whole brain. Right hippocampus/parahippocampus-seeded RSFC in right middle/inferior temporal gyrus significantly increased in the POCD group (P < 0.001, GRF correction, cluster size > 26) as compared to that in the non-POCD group (Figure 3).

### Correlations Between RSFC and NPTs Performance

**Figure 4:**
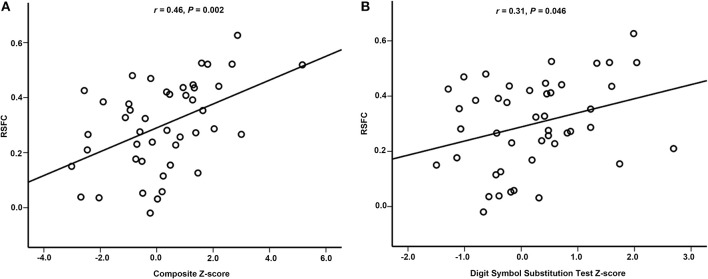
Partial correlation between RSFC and neuropsychological performance. **(A)** There was partial correlation between RSFC and composite *Z*-score. **(B)** There was partial correlation between RSFC and Digit Symbol Substitution Test *Z*-score. RSFC, resting-state functional connectivity.

After adjusting for education duration, smoking, and sex, positive correlations were found between RSFC and composite *Z*-scores (*r* = 0.46, 95% CI [0.234, 0.767], *P* = 0.002, Partial Correlation) or Digit Symbol Substitution Test *Z*-scores (*r* = 0.31, 95% CI [0.068, 0.643], *P* = 0.046, Partial Correlation), respectively (Figure 4).

## Discussion

The present investigation provided several new insights into pre-operative cognition impairment in elderly patients who developed early POCD after major non-cardiac surgery. The key findings included: 1) POCD patients displayed a cluster with higher pre-operative ReHo in the right hippocampus/parahippocampus as compared with non-POCD patients. 2) Pre-operative RSFC between right hippocampus/parahippocampus and right middle/inferior temporal gyrus was enhanced in POCD patients. 3) The right hippocampus/parahippocampus-seeded RSFC in right middle/inferior temporal gyrus positively correlated with composite *Z*-scores or Digit Symbol Substitution Test Z-score. Taken together, the present results demonstrated that pre-existing alterations of intrinsic brain activity might exist in elderly patients who developed early POCD.

The hippocampus/parahippocampus lies in the MTL and belongs to limbic system. Hippocampus/parahippocampus plays a fundamental role in extensive cognitive domains, such as visuospatial processing, episodic memory, contextual associations, spatial working memory, and long-term memory (27, 28). During memory processing, encoding usually occurs in a process with attention exploited (29), and the consolidation can happen in sleep or a resting-state without a specific task (30). Hippocampus/parahippocampus is also an element of DMN (31). DMN performs a critical function not only in spontaneous cognition (e.g., mind wandering, daydreaming), but also in the performance of cognitively demanding tasks (32). The hippocampus/parahippocampus is the earliest atrophic brain region in Alzheimer's disease pathological process even in the presymptomatic stage. Before atrophy, patients with mild cognitive impairment (MCI) had already revealed some resting-state functional abnormality. As compared with cognitively healthy subjects, increased ReHo was observed in the bilateral hippocampus/parahippocampus in MCI patients without lacunar infarctions (33).

The hippocampus/parahippocampus also work as parts of MTL memory system which consist of the hippocampus, entorhinal cortex, perirhinal, and parahippocampal cortices (34). The MTL memory system has a complicated relationship within its own components and with the lateral temporal lobe (35). RSFC studies have shown that the coupling of hippocampus/parahippocampus and right middle/inferior temporal gyrus is of involvement in quite a few cognitive processes, such as semantic memory, visual perception, as well as attention and working memory (36). In healthy subjects, the anterior hippocampus shares RSFC with the entorhinal cortex and a lateral temporal network including middle/inferior temporal gyrus (37). An fMRI study in patients undergoing anterior temporal lobe resection revealed that post-operative working memory is dependent on the functional capacity reserve of the right hippocampus (38). These data from both healthy subjects and patients add evidence to the hypothesis that right hippocampus/parahippocampus and right middle/inferior temporal gyrus work as a part of the MTL memory system. In the present study, pre-operative RSFC between right hippocampus/parahippocampus and right middle/inferior temporal gyrus significantly increased in POCD patients. The POCD patients exhibited a significant decrease in global cognition, especially in semantic memory, working memory and attention, which had a lot of overlap with the function of hippocampus/parahippocampus and middle/inferior temporal gyrus.

RSFC between right hippocampus/parahippocampus and right middle/inferior temporal gyrus positively correlated with Digit Symbol Substitution Test *Z*-scores or composite *Z*-scores in the present study. The Digit Symbol Substitution Test can be used to assess several domains of cognitive function, including executive function, attention, and working memory (39). The composite *Z*-scores represents the changes in global cognitive dysfunction. The results suggest that patients with higher pre-operative RSFC between right hippocampus/parahippocampus and right middle/inferior temporal gyrus were prone to deterioration of post-operative cognitive function. This is consistent with previous studies about cognitive alterations in normal aging and Alzheimer's disease, in which increased functional connectivity is interpreted as an attempt to maintain cognitive performance (40). Previous studies have demonstrated the existence of age-related compensatory mechanisms for cognitive preservation. These age-related functional changes in brain activation patterns allow for cognitive performance to be preserved (41). When the damaged cognitive preservation was challenged by a cognitive task such as increasing working memory load, the degree centrality and local coherence in the left dorsal posterior cingulate cortex (dPCC) increased, which was inversely associated with global cognitive outcomes (42). It has also been demonstrated that cognitive function such as psychomotor speed would be improved after cerebral arterial perfusion increased (43). Based on previous and present results, we speculated that POCD patients might have subtle cognitive decline and functional connectivity alterations before surgery. These patients do not have clinically detectable cognitive symptoms because of the existence of compensatory mechanisms. However, cognitive symptoms may become apparent after the stressors of surgery and anesthesia.

A major limitation to this study was the small sample size, which was not subjected to a priori power calculation. The difficulty of patient recruitment might contribute to a relatively small sample size which could result in potential type II error. The second limitation was that post-operative cognitive function was examined at discharge without screening post-operative delirium (POD). POD could not be completely ruled out in the present study. Therefore, more of a caveat should be placed on the results. Finally, because of the drop-out at 3 months after surgery, the number of patients was not enough for analyzing fMRI data at 3 months. The association between pre-operative brain functional alterations and cognitive decline at 3 months requires further investigation.

## Conclusion

In conclusion, the present investigation demonstrates the difference in pre-operative ReHo in the right hippocampus/parahippocampus between POCD patients and non-POCD patients. The pre-operative ReHo-seeded RSFC in right middle/inferior temporal gyrus increases in POCD patients, and positively correlates with alterations of cognitive function. The results suggest that premorbid alterations of regional spontaneous activity and functional connectivity exist in elderly patients who develop early POCD after major non-cardiac surgery. The neural mechanism that patients with abnormal pre-operative spontaneous activity are susceptible to POCD remains an area of further study.

## Data Availability Statement

The raw data supporting the conclusions of this manuscript will be made available by the authors, without undue reservation, to any qualified researcher.

## Ethics Statement

This study has been approved by the Ethics Committee of Huadong Hospital affiliated with Fudan University with the Approved Number of 20150056. This study was registered before patient enrollment at http://www.chictr.org.cn with the identifier of ChiCTR-DCD-15006096 on 16th March 2015. Informed consent was obtained from every participant after full explanation of the protocol. Authors followed the Declaration of Helsinki principles.

## Author Contributions

XZ was one of the first author of the manuscript, who helped design the study, conduct the study, analyze the data, draft, and revise the manuscript. HL contributed equally to XZ. YL was responsible for the statistical work, also helped revise this manuscript. ZZ helped review the manuscript. XS, QL, and WW helped review the manuscript. ZW helped design the study. ZJ helped collect the data. LY helped design the study. GL contributed equally to WG. WG helped design the study, conduct the study, analyze the data, draft, and revise the manuscript.

### Conflict of Interest

The authors declare that the research was conducted in the absence of any commercial or financial relationships that could be construed as a potential conflict of interest.
