# Supplementary material for: Premorbid Alterations of Spontaneous Brain Activity in Elderly Patients With Early Post-operative Cognitive Dysfunction: A Pilot Resting-State Functional MRI Study
Source: Front Neurol. 2019 Oct 9;10:1062. doi: 10.3389/fneur.2019.01062 (PMC6794447; doi:10.3389/fneur.2019.01062)
Supplement: Supplementary file 1 [file Table_1.DOC]

**Supplemental Table．**

Baseline and difference of cognitive function

| NPTs | POCD (n=13) | Non-POCD (n=32) | *P-*value |
| --- | --- | --- | --- |
| **MMSE** |  |  |  |
| Preoperative, median (IQR) | 27.0 (26.0-28.5) | 27.5 (27.0-29.0) | 0.47 |
| Postoperative, median (IQR) | 27.0 (23.5-28.5) | 28.0 (27.0-29.0) | 0.13 |
| Difference, mean (SD) | 1.11 (2.00) | 0.46 (1.48) | 0.35 |
| **VFT** |  |  |  |
| Preoperative, mean (SD) | 14.9 (4.0) | 15.4 (3.0) | 0.63 |
| Postoperative, mean (SD) | 14.0 (4.2) | 16.9 (3.9) | 0.021 |
| Difference, median (IQR) | 1.15 (0.23-1.30) | 0.23 (-0.38-0.76) | 0.021 |
| **DSF** |  |  |  |
| Preoperative, median (IQR) | 7.0 (6.5-8.0) | 8.0 (7.0-8.8) | 0.25 |
| Postoperative, median (IQR) | 7.0 (5.0-8.0) | 8.0 (8.0-8.0) | 0.001 |
| Difference, mean (SD) | 1.68 (1.15) | 0.55 (1.53) | 0.015 |
| **DSB** |  |  |  |
| Preoperative, median (IQR) | 4.0 (3.5-5.5) | 3.0 (3.0-4.0) | 0.07 |
| Postoperative, median (IQR) | 4.0 (3.0-4.0) | 4.0 (3.0-4.0) | 0.27 |
| Difference, median (IQR) | 0.75 (0.00-1.49) | 0.00 (-0.75-0.56) | 0.005 |
| **DSST** |  |  |  |
| Preoperative, median (IQR) | 23.0 (19.5-28.0) | 29.5 (20.0-37.0) | 0.16 |
| Postoperative, median (IQR) | 21.0 (16.0-27.0) | 30.0 (23.0-38.8) | 0.002 |
| Difference, median (IQR) | 0.93 (0.49-1.81) | -0.18 (-0.62-0.27) | ＜0.001 |
| **TMT-A** (seconds) |  |  |  |
| Preoperative, median (IQR) | 46.0 (38.5-65.0) | 45.5 (32.8-73.8) | 0.75 |
| Postoperative, median (IQR) | 53.0 (43.5-90.0) | 40.5 (32.3-61.0) | 0.026 |
| Difference, median (IQR) | 0.60 (-0.07-3.16) | -0.07 (-1.00-0.49) | 0.021 |
| **Composite Z-score, mean (SD)** | 2.99 (1.73) | 0.27 (1.13) | ＜0.001 |

Supplemental Table 1: Lists the baseline, postoperative and difference of NPTs. Note: Difference is the cognitive change between preoperative and postoperative score, which is displayed as Z-score of a single NPT in form of mean ± SD or median with IQR. Composite Z-score is the sum of a patient’s Z-score of all 6 NPTs. NPTs, neuropsychological tests; MMSE, Mini-mental state Examination; IQR, interquartile range; SD, standard deviation; VFT, Verbal Fluency Test; DSF, Digit Span Forwards; DSB, Digit Span Backwards; DSST, Digit Symbol Substitution Test; TMT-A, Trail Making Test part A.
